# Anomalous tensile response of bacterial cellulose nanopaper at intermediate strain rates

**DOI:** 10.1038/s41598-020-72153-w

**Published:** 2020-09-17

**Authors:** Alba Santmarti, Hon Wah Liu, Natalia Herrera, Koon-Yang Lee

**Affiliations:** Department of Aeronautics, Imperial College London, South Kensington Campus, London, SW7 2AZ UK

**Keywords:** Engineering, Materials science

## Abstract

Nanocellulose network in the form of cellulose nanopaper is an important material structure and its time-dependent mechanical response is crucial in many of its potential applications. In this work, we report the influences of grammage and strain rate on the tensile response of bacterial cellulose (BC) nanopaper. BC nanopaper with grammages of 20, 40, 60 and 80 g m^−2^ were tested in tension at strain rates ranging from 0.1% s^−1^ to 50% s^−1^. At strain rates $\le$ 2.5% s^−1^, both the tensile modulus and strength of the BC nanopapers stayed constant at ~ 14 GPa and ~ 120 MPa, respectively. At higher strain rates of 25% s^−1^ and 50% s^−1^ however, the tensile properties of the BC nanopapers decreased significantly. This observed anomalous tensile response of BC nanopaper is attributed to inertial effect, in which some of the curled BC nanofibres within the nanopaper structure do not have enough time to uncurl before failure at such high strain rates. Our measurements further showed that BC nanopaper showed little deformation under creep, with a secondary creep rate of only ~ 10^–6^ s^−1^. This stems from the highly crystalline nature of BC, as well as the large number of contact or physical crosslinking points between adjacent BC nanofibres, further reducing the mobility of the BC nanofibres in the nanopaper structure.

## Introduction

Cellulose nanopaper, a fibrous network consisting of cellulose nanofibres connected by physical entanglements and hydrogen bonding, is an interesting and important material structure. Despite its similar structure to conventional paper, the large number of contact or physical crosslinking points between adjacent cellulose nanofibres give rise to the superior mechanical properties of cellulose nanopaper over conventional paper^1^. The tensile modulus and strength of a 60 g m^−2^ cellulose nanopaper were reported to be ~ 12 GPa and ~ 135 MPa, respectively^2^. At the same grammage, conventional paper possesses a tensile modulus of only ~ 3 GPa and a tensile strength of ~ 40 MPa. The global demand for cellulose nanofibres in 2018 was 10,000 tons and this demand is estimated to increase to more than 70,000 tons by the year 2030^3^, with the highest volume in the paper and packaging sectors, whereby cellulose nanopaper is anticipated to be an important material structure^4–10^. In addition to this, cellulose nanopaper is often explored for various advanced engineering applications, including membrane for water filtration^11–13^, substrate for flexible electronics^14,15^ and more recently as two-dimensional reinforcement for polymers^16–19^.

Considering the many potential applications of cellulose nanopaper, it is likely that cellulose nanopaper will not be subjected to loading conditions only in the quasi-static regime (strain rates of ~ 10^–5^–10^–2^ s^−1^)^20^. When used in structural or packaging applications for example, cellulose nanopaper would be under constant stress for an extended period of time and/or subjected to dynamic loading. Furthermore, the commercial production of cellulose nanopaper is anticipated to utilise web processing technique, such as roll-to-roll manufacturing^21^. In this context, the time-dependent mechanical response of cellulose nanopaper is important as it is closely connected to the web stress behaviour at different transport velocities^22^. Production speed in roll-to-roll manufacturing is also crucial as it controls the web tension at which the fibre network is subjected to^23^. Cellulose nanopaper could be subjected to high pulling speed in the calendaring step. Whilst this is currently not known for cellulose nanopaper, conventional paper is typically subjected to speeds of up to 1,200 m min^−1^^24^. It can therefore be anticipated that the mechanical properties of cellulose nanopaper at strain rates beyond the quasi-static regime are of interest to assess the possibility of high-speed cellulose nanopaper manufacturing, as well as the potential of cellulose nanopaper in industrially relevant loading conditions.

Here in this work, we report our findings on the anomalous tensile response of bacterial cellulose (BC) nanopaper at intermediate strain rates of up to 50% s^−1^, as well as its viscoelastic and creep properties. BC is used in this work as it is an ultrapure form of cellulose nanofibre with uniform fibre diameter of *ca.* 50 nm^25^, without impurities such as hemicellulose or traces of lignin that are often present in wood-derived cellulose nanofibres^26^. A previous study of ours^16^ also showed that the production time of cellulose nanopaper could be reduced by decreasing the grammage of the nanopaper to be produced, which could be beneficial in the large scale production of cellulose nanopaper. Therefore, the time-dependent tensile response of BC nanopaper is also discussed in terms of its grammage.

## Results and discussion

**Figure 1:**
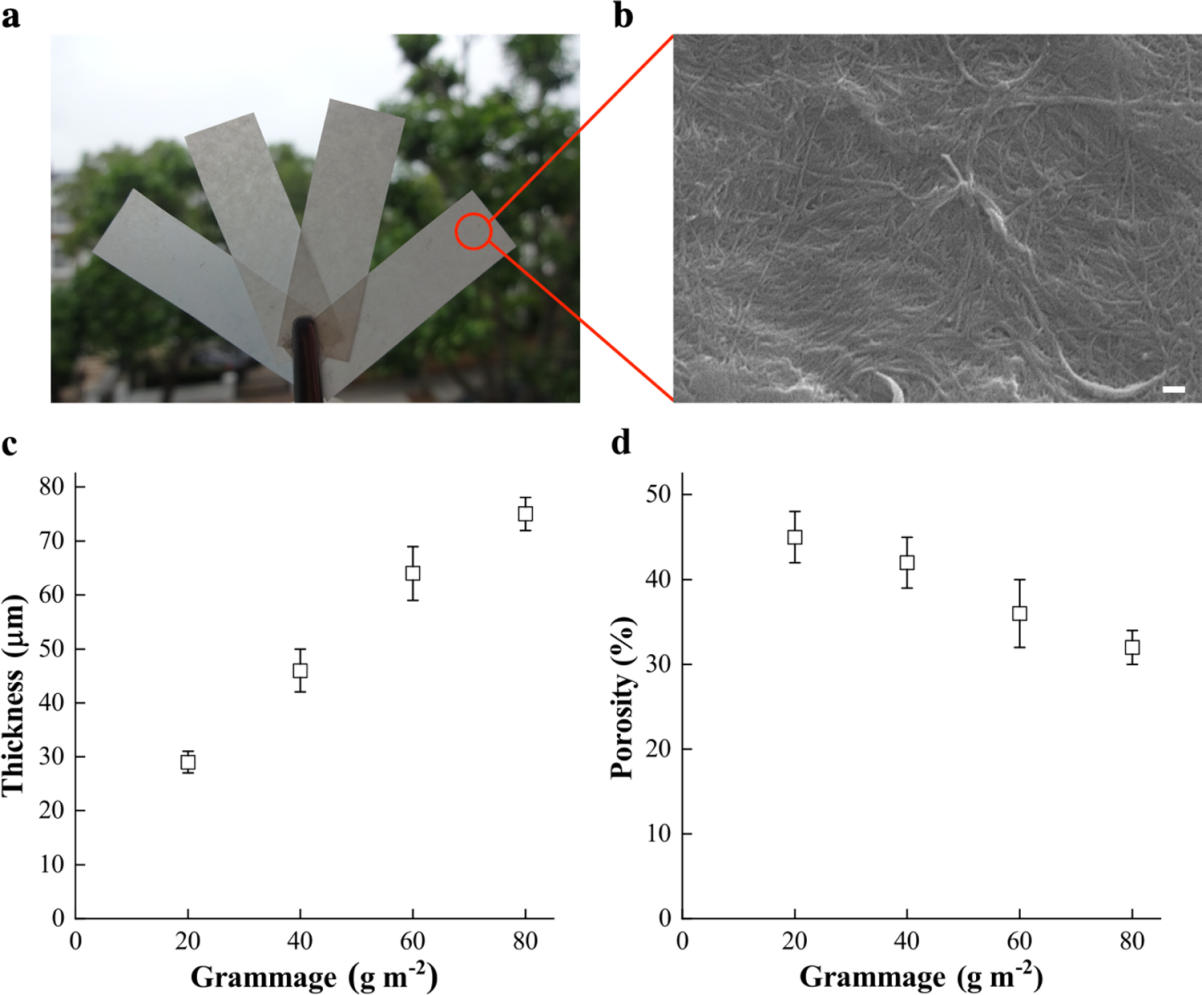
(**a**) Visual appearance of the model BC nanopapers fabricated. From left to right: 20 gsm, 40 gsm, 60 gsm and 80 gsm, respectively. (**b**) Scanning electron micrographs showing the morphlogy of a BC nanopaper. The scale bar denotes 300 nm. (**c**) Thickness and (**d**) porosity of the BC nanopapers as a function of grammage.

Figure 1a shows the visual appearance of the BC nanopapers produced and the surface morphology of a BC nanopaper is shown in Fig. 1b. It can be seen from this figure that BC nanopaper possessed a web-like structure consisting of a random network of cellulose nanofibres. The thickness and porosity of the model BC nanopapers are presented in Fig. 1c,d, respectively. Model BC nanopaper with the lowest grammage of 20 gsm possessed a thickness of only 30 μm and a porosity of 45%. On the other hand, BC nanopaper with the highest grammage of 80 gsm possessed a thickness of 75 μm and a lower porosity of 32%. The reduction in nanopaper porosity with increasing nanopaper grammage can be attributed to the improved packing efficiency of cellulose nanofibres when the grammage increases^16^.

**Figure 2:**
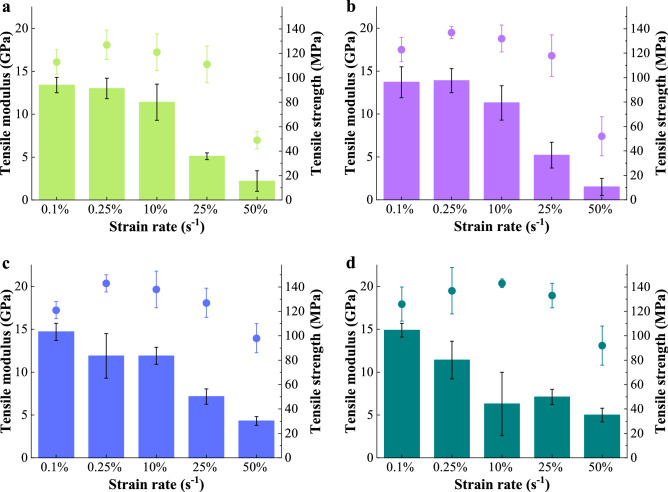
The tensile properties of (**a**) 20 g m^−2^, (**b**) 40 g m^−2^, (**c**) 60 g m^−2^ and (**d**) 80 g m^−2^ BC nanopapers tested at different strain rates. The columns correspond to tensile modulus and the circular symbols denote tensile strength. The strain rates of 0.1% s^−1^, 0.25% s^−1^, 10% s^−1^, 25% s^−1^ and 50% s^−1^ correspond to crosshead displacement speeds of 1 mm min^−1^, 30 mm min^−1^, 100 mm min^−1^, 300 mm min^−1^ and 600 mm min^−1^, respectively.

The model BC nanopapers were tested in uniaxial tension at five different crosshead displacement speeds ranging from 1 mm min^−1^ to 600 mm min^−1^, which corresponded to strain rates of 0.1% s^−1^ to 50% s^−1^, respectively. Figure 2 summarises the measured tensile properties of BC nanopapers at different grammage tested at different strain rates (see Fig. S1 and Table S1 in the supplementary information for their representative tensile stress–strain curves and the tabulated tensile properties, respectively). The tensile modulus and strength of BC nanopapers were measured to be 13.5–14.9 GPa and 113–125 MPa, respectively, under quasi-static testing condition (crosshead displacement speed = 1 mm min^−1^, strain rate = 0.1% s^−1^). The slightly higher tensile properties of BC nanopapers at higher grammage could be attributed to their lower porosity. Nevertheless, the quasi-static tensile properties of the BC nanopapers in this work are in good agreement with the tensile properties of cellulose nanopapers reported in the literature, all of which were tested under similar strain rates^27^.

When BC nanopaper was tested at higher strain rates however, the tensile properties of the nanopaper decreased significantly. At a strain rate of 50% s^−1^, the tensile modulus and strength of 60 and 80 gsm BC nanopapers reduced from ~ 14.9 GPa and ~ 125 MPa, respectively (quasi-static), to only ~ 5.0 GPa and ~ 80 MPa, respectively. This decrease is particularly drastic for 20 and 40 gsm BC nanopapers, whereby the tensile modulus and strength decreased to only ~ 2.0 GPa and ~ 50 MPa, respectively, when subjected to a strain rate of 50% s^−1^. We attribute the different tensile response between the lower and higher grammage nanopapers to the difference in the stress transfer efficiency of the BC nanopapers. The efficiency of stress transfer within a random fibre network is related to its mean coverage ($\overline{c}$), which is defined as the number of fibres covering a point in the plane of support of the fibre network^28^. The stress-transfer efficiency function $\left( \varphi \right)$ is given by^29^:

1 $$\varphi = \left( {1 - \left( {1 + \overline{c}} \right)e^{{ - \overline{c}}} } \right)\left( {1 + \frac{{\gamma + \log \left( {\overline{c}} \right) - (Chi\left( {\overline{c}} \right) + Shi\left( {\overline{c}} \right)}}{{e^{{\overline{c}}} - 1}}} \right)\theta$$

where $\theta$ is a constant that follows the inequality $0 < \varphi \le \theta$, $\gamma$ is the Euler’s constant, ${\text{Chi}}\left( z \right)$ and ${\text{Shi}}\left( z \right)$ are the hyperbolic cosine and sine integrals. The mean coverage of a two–dimensional fibre network ($\overline{c}$) is further given by^30^:

2 $$\overline{c} = \frac{{\overline{\beta }\omega }}{{\rho_{L} }}$$

where $\overline{\beta }$ corresponds to the average grammage of the fibre network, $\omega$ corresponds to the fibre width and $\rho_{L}$ is the linear density of the fibre. As the BC nanopapers prepared in this work were produced from the same BC source, the values of $\omega$ and $\rho_{L}$ are the same for all samples. It can therefore be inferred from Eqs. (1) and (2) that higher grammage BC nanopaper will possess higher coverage and consequently, better stress transfer between adjacent BC nanofibres within the nanopaper structure compared to their lower grammage counterpart. As a result, the load bearing capability of 60 and 80 gsm BC nanopapers is better at higher strain rates compared to 20 and 40 gsm BC nanopapers.

Nevertheless, the decrease in the tensile properties with increasing strain rates is in direct contradiction with the strain rate sensitivity of common materials, which typically exhibit strain rate hardening behaviour. The tensile moduli and strengths of polymers^31^, metals and alloys^32^, borosilicate glass and glass fibres^33^ increase with increasing strain rates, albeit to varying degrees. Conventional paper is also a material structure that exhibits strain rate hardening behaviour. The works of Rance^34^, as well as Andersson and Sjöberg^35^ have shown that the tensile properties of paper increase with increasing strain rates. More recent work by Kouko and Retulainen^36^ showed that this strain rate hardening behaviour can also be extended to wet paper with a solid content of between 40 and 60 wt%.

We therefore first ascertain whether the BC nanopapers tested were in a uniform state of uniaxial stress. This can be confirmed by comparing the time scale of the uniaxial tensile test at the highest strain rate to the time taken for the stress wave to make one roundtrip along the test specimen ($T_{{{\text{roundtrip}}}}$)^37^:

3 $$T_{{{\text{roundtrip}}}} = \frac{2L}{{C_{{{\text{wave}}}} }}$$

where $L$ corresponds to the (exposed) length of the specimen (20 mm in this work) and $C_{{{\text{wave}}}}$ denotes the one-dimensional wave speed in the specimen, which can be calculated from the Young’s modulus ($E$) and envelope density ($\rho_{e}$) of the test specimen:

4 $$C_{{{\text{wave}}}} = \sqrt {\frac{E}{\rho_{e} }}$$

Using the measured Young’s modulus at the highest strain rate (to obtain the worst-case scenario), we obtained $T_{{{\text{roundtrip}}}}$ values in the order of ~ 10^–2^ ms (see Table 1). The time scale of our uniaxial tensile test conducted at 50% s^−1^, on the other hand, was three orders of magnitude higher at 30 ms. This implies that the test specimens have achieved a state of uniform stress during uniaxial tensile test as a large number of stress wave reverberations have been achieved during the test.

**Table 1:** T_roundtrip_ values calculated for BC nanopapers with different grammages tested at 50% s^−1^ strain rate.

Grammage (g m^−2^)	ρ_e_ (g cm^−3^)	T_roundtrip_ (× 10^–2^ ms)
20	0.82 ± 0.05	2.44 ± 0.69
40	0.87 ± 0.05	3.04 ± 1.06
60	0.94 ± 0.05	1.87 ± 0.12
80	1.02 ± 0.03	1.81 ± 0.15

**Figure 3:**
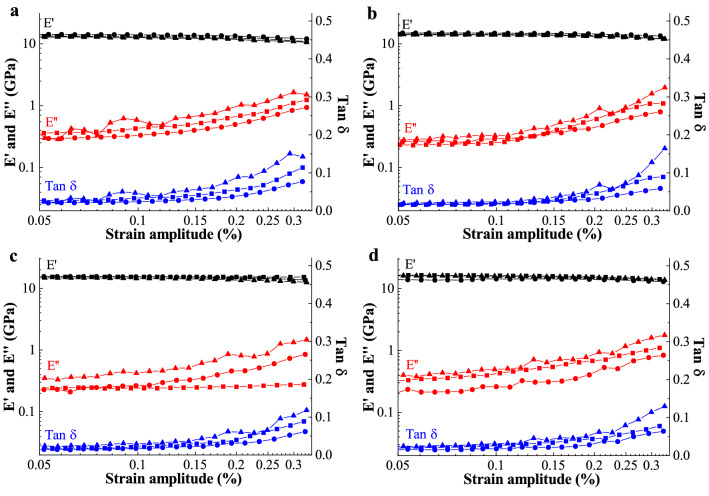
Viscoelastic properties of (**a**) 20 g m^−2^, (**b**) 40 g m^−2^, (**c**) 60 g m^−2^ and (**d**) 80 g m^−2^ BC nanopapers. E′, E′′ and tan ${\updelta }$ denote the storage modulus, loss modulus and mechanical loss factor, respectively. ▲ = 0.1 Hz; ■ = 1 Hz; ● = 10 Hz.

As the time-dependent mechanical response of a polymeric material is closely linked to its viscoelastic behaviour, the viscoelastic properties of BC nanopaper were further studied to elucidate the anomalous tensile response of BC nanopaper at intermediate strain rates. Dynamic mechanical analysis of the BC nanopaper was performed in tensile mode. A pre-load of 3 N was applied prior to applying a periodic deformation to avoid buckling of the test specimen during the compressive part of the stress–strain cycle. Figure 3 summarises the storage modulus (*E*′), loss modulus (*E*″) and the mechanical loss factor (tan $\delta$) measured at 0.1, 1 and 10 Hz, respectively, as a function of strain amplitude for BC nanopapers of different grammage. It can be seen from this figure that the dynamic mechanical response of the BC nanopapers is similar, irrespective of their grammage. The *E*′ of the BC nanopaper was found to be insensitive to the applied oscillatory frequencies and strain amplitudes. These results corroborate with the lack of strain rate hardening response of BC nanopaper.

**Figure 4:**
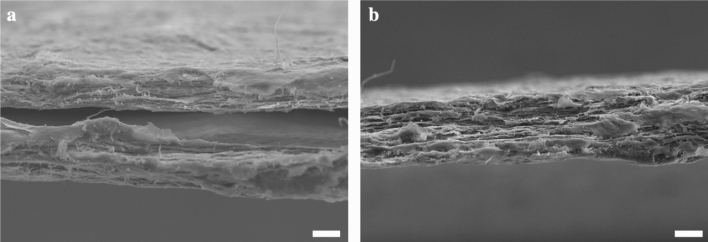
Exemplary fracture surfaces of an 80 gsm BC nanopaper tested at (**a**) 0.1% s^−1^ and (**b**) 50% s^−1^. The scale bars represent 10 µm.

The tan $\delta$ of the BC nanopaper, on the other hand, was found to decrease with increasing applied oscillatory frequency (see Fig. 3). At an oscillation strain amplitude of 0.3% and a frequency of 0.1 Hz, BC nanopaper possess a tan $\delta$ value of ~ 0.1. When tested at an oscillatory frequency of 10 Hz and at the same oscillation strain, the tan $\delta$ value of the BC nanopaper decreased to only ~ 0.05. This viscoelastic response suggests fibre slippage or fibre debonding at high strain amplitudes or oscillatory frequencies. This is also congruent with the fracture morphology of the BC nanopaper (Fig. 4), which showed signification defibrillation and this also suggests fibre slippage or fibre debonding instead of fibre re-orientation and fracture. It is also worth mentioning that in a fibre network, interfibre friction (which stems from segments of exposed fibres between the contact points of the fibre network) typically contributes to an increase in the value of tan $\delta$^38^. As the value of tan $\delta$ was found to decrease with increasing strain amplitudes or oscillatory frequencies, it is further deduced that interfibre friction in the BC nanopaper structure is limited, presumably due to the large number of contact or physical crosslinking points between adjacent BC nanofibres within the nanopaper.

Creep measurement is also a good indicator of the time-dependent mechanical properties of a material. Usually, viscoelastic materials slowly deform (creep strain) when they are under constant stress for an extended period of time^39^. The total creep strain can be divided into four stages: initial strain, primary creep, secondary creep and tertiary creep^40^. Initial strain corresponds to the immediate elastic deformation occurring during initial loading. This is then followed by primary creep, characterised by a decrease in the deformation rate. Primary creep is considered as a delayed response of the material to the applied stress in which the structure of the material adjusts itself to the following steady-state secondary creep stage. During this secondary creep stage (also called stationary creep), the material flows in a viscous (plastic) manner and the creep rate stays constant. In the last stage, known as tertiary creep, the rate of deformation increases again until the material fails^41^. Initial strain and primary creep are considered to be fully recoverable whereas secondary and tertiary creep are nonrecoverable^39^.

**Figure 5:**
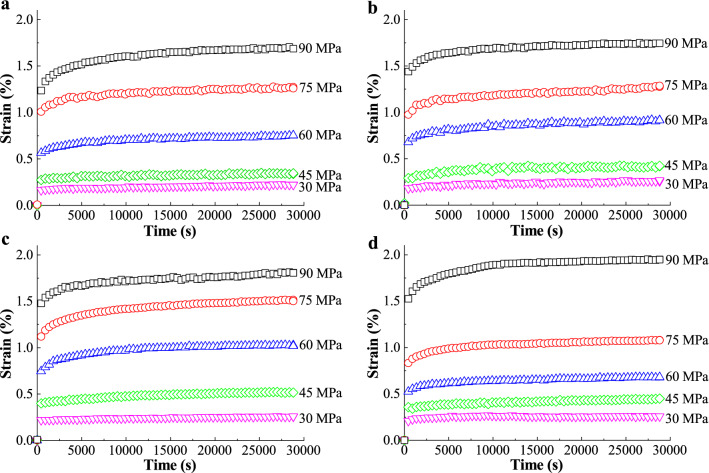
Representative creep curves of (**a**) 20 g m^−2^, (**b**) 40 g m^−2^, (**c**) 60 g m^−2^ and (**d**) 80 g m^−2^ BC nanopapers.

Measured creep curves, i.e. $\varepsilon = f\left( t \right)$, of BC nanopapers at different grammage subjected to various initial stresses are plotted in Fig. 5. When the initial stress applied to the nanopaper was below 40% of its ultimate strength (e.g. 30 and 45 MPa), the creep curves exhibited an elastic response since most of the strain corresponded to elastic strain and creep was almost non-existent. However, when BC nanopaper was loaded at higher stresses (75 MPa and 90 MPa), the BC nanopaper exhibited primary creep deformation, which can be attributed to fibre slippage and fibre-debonding. As the strain deformation rate decreased, it levelled to a steady state creep rate typical of secondary creep. Secondary creep rates for BC nanopapers at different grammage subjected to various initial stresses are presented in Table 2. Most materials exhibit a linear dependency, in which the secondary creep rate increases at higher stresses^42^. However, the secondary creep rate in BC nanopaper is relatively independent of the initial applied stress. The stationary creep rate obtained for BC nanopaper is in the order of ~ 10^–6^ s^−1^, whereas paper made from bleached kraft pulp under an initial stress equivalent to 75% of its tensile strength exhibited a stationary creep an order of magnitude higher at ~ 10^–5^ s^−1^^43^. Typical viscoelastic polymers such as polyethylene tested at initial stress of 20 MPa exhibited a stationary creep rate two orders of magnitude higher at ~ 10^–4^ s^−1^^44^. Since the amount of creep deformation and creep rate depend on the stability of the fibre bonds and on the stored energy of the system, this implies that the BC nanofibres are firmly bonded through hydrogen bonds at the contact points between adjacent fibres. This resulted in restricted viscous flow and therefore do not exhibit much permanent deformations (secondary creep)^45^.

**Table 2:** Secondary creep rates of BC nanopapers at different gramamge as a function of initial applied stress.

Initial applied stress (MPa)	Secondary creep rate / × 10^–6^ s^−1^			
	20 g m^−2^	40 g m^−2^	60 g m^−2^	80 g m^−2^
30	2.8 ± 0.1	1.7 ± 0.1	0.8 ± 0.1	0.2 ± 0.1
45	1.1 ± 0.1	1.1 ± 0.2	1.8 ± 0.1	1.9 ± 0.1
60	1.9 ± 0.1	1.6 ± 0.6	2.0 ± 0.2	2.1 ± 0.1
75	2.9 ± 0.1	4.8 ± 0.1	4.6 ± 0.1	2.6 ± 0.1
90	3.5 ± 0.2	2.4 ± 0.2	5.2 ± 0.2	2.6 ± 0.1

Creep rates are calculated from the slope of the creep curve (Fig. 5) in the time interval of between 10,000 and 28,800 s.

Our findings suggest that the limited secondary creep deformation, due to the large number of contact or physical crosslinking points between adjacent BC nanofibres combined with the highly crystalline nature of BC, which was measured to be ~ 90% based on our previous studies^25^, is the main cause for the anomalous tensile response of BC nanopaper at intermediate strain rates. When subjected to strain rates beyond the quasi-static regime, some of the BC nanofibres within the nanopaper structure might not have enough time to respond to the applied load as some of the nanofibres are curled (see Fig. 1b)^46^. The large number of contact points further limits nanofibre uncurling and nanofibre reorientation within the nanopaper structure, consistent with our fractographic analysis (Fig. 4). As a result, the nanopaper fails due to inertial effect. This corroborates with the tensile stress–strain curves of the BC nanopaper (see Fig. S1 in the supplementary information), whereby little signs of plastic deformation were observed when subjected to higher strain rates.

## Conclusions

We investigated the tensile response of BC nanopaper from quasi-static to intermediate strain rates. It was found that BC nanopaper does not exhibit strain rate hardening behaviour. Instead, the tensile properties of BC nanopaper decreased at intermediate strain rates. This anomalous tensile response was also found to be independent of the grammage of the nanopaper. We attribute this lack of strain rate sensitivity to the low secondary creep rate (~ 10^–6^ s^−1^) of BC nanopaper, which stems from the large number of contact or physical crosslinking points between adjacent BC nanofibres and the high degree of crystallinity of BC. This limited the uncurling and reorientation of the BC nanofibres within the nanopaper structure, constricting plastic deformation at higher strains. At strain rates above 25% s^−1^, it is postulated that the BC nanofibres in the nanopaper structure could not react to the applied load. As a result, the nanofibres fail in their curled state. Whilst the lack of strain rate hardening in BC nanopaper is not desirable, particularly as two-dimensional reinforcement for composite applications or high-speed BC nanopaper processing, the low secondary creep rate and limited secondary creep deformation suggest that BC nanopaper could be an attractive material for applications where creep should be minimised, such as packaging film applications.

## Methods

### Materials

BC was purchased from a commercial retailer (Xiangsun Ltd., Lugang Township, Changhua County, Taiwan) in the form of nata de coco cubes prior to bottling in a sugary syrup. These nata de coco cubes contain 2.5 wt% BC (dry basis). Sodium hydroxide pellets (AnalaR NORMAPUR, purity = 98.5%) were purchased from VWR International Ltd. (Lutterworth, UK).

### Purification of BC

For each batch of 200 g of nata de coco, the cubes were suspended with 4 L of de-ionised water and heated to 80 °C. Once the desired temperature was reached, 16 g of NaOH pellets were added and the nata de coco cubes-in-water suspension was left to stir at 80 °C for 2 h in order to remove any remaining microorganism or soluble polysaccharides. After this step, the suspension was left to cool to room temperature, prior to pouring the purified nata de coco cubes onto a metal sieve to drain away any excess liquid. The purified nata de coco cubes were further rinsed with de-ionised water until neutral pH was attained, followed by blending (Duronic BL10) in 0.5 L of de-ionised water using a blender operating at maximum power output of 1,000 W for 2 min to produce a homogeneous BC suspension. The BC suspension was then centrifuged at 6,000 rpm for 10 min to remove the excess water, producing a BC aqueous gel with a consistency (i.e. solid content) of 2 wt%. The BC aqueous gel was stored in a 4 °C fridge prior to subsequent use.

### Manufacturing of BC nanopapers

To prepare the BC nanopaper, an appropriate amount of the purified BC gel (based on the required grammage of the BC nanopaper to be produced) was dispersed in 0.5 L of deionised water using a blender to create a homogenous BC-in-water suspension. This suspension was then vacuum filtered onto a filter paper (Grade 413 cellulose filter paper, VWR International Ltd.) using a Büchner funnel. The wet BC filter cake was then carefully removed from the used filter paper and placed on a glass plate, followed by drying at 70 ºC for 3 h. Extra care was taken to avoid trapping air bubbles in between the glass plate and the wet BC filter cake.

### Characterisation of the manufactured BC nanopapers

#### Scanning electron microscopy (SEM)

The surface and fracture morphology of the BC nanopapers were investigated using a large chamber SEM (S-3700 N, Hitachi, Tokyo, Japan). The samples were glued onto aluminium stubs using epoxy resin containing conductive carbon black. All samples were then coated with Au (40 mA, 30 min) using an auto sputter coater (Agar Scientific, Stansted, UK) prior to SEM. The accelerating voltage used was 15 kV.

### Density and porosity of the BC nanopapers

The thickness of the BC nanopapers was measured using a digital micrometer (Mitutoyo MDC Lite, RS Components Ltd., Dunstable, UK). With the thickness and projected area known, the bulk volume of the nanopaper was calculated. The envelope density ($\rho_{e}$) of the BC nanopapers was then obtained by taking the ratio between the mass and the calculated bulk volume of the sample. The true density ($\rho_{t}$) of the BC nanopapers was taken to be 1.5 g cm^−3^ based on our previous measurement^27^. The porosity (*P*) of the BC nanopapers was calculated using:

5 $$P\left[ \% \right] = \left( {1 - \frac{{\rho_{{\text{e}}} }}{{\rho_{{\text{t}}} }}} \right) \times 100$$

### Tensile properties of the BC nanopapers

Prior to tensile test, the manufactured BC nanopapers were prepared following our previously described work^19,26^. Briefly, BC nanopapers were cut into rectangular test specimens with an overall length of 35 mm and a width of 5 mm using a Zwick/Roell ZCP 020 manual cutting press (Zwick Testing Machines Ltd., UK). All the test specimens were then secured onto 140 gsm paper testing cards using two-part cold curing epoxy resin (Araldite 2011, Huntsman Advanced Materials, UK). This was to avoid the clamps of the tensile tester from damaging the ends of the test specimens, potentially leading to earlier onset failure within the gripping zone. After securing the test specimens onto the testing cards, the exposed length of the rectangular test specimens was 20 mm.

Tensile test was performed using an Instron universal tester (Model 5,969, Instron, High Wycombe, UK) equipped with a 10 kN load cell. The test was conducted at crosshead displacement speeds of 1 mm min^−1^, 30 mm min^−1^, 100 mm min^−1^, 300 mm min^−1^ and 600 mm min^−1^, which corresponded to strain rates of 0.1% s^−1^, 0.25% s^−1^, 10% s^−1^, 25% s^−1^ and 50% s^−1^, respectively. Two points were marked on the surface of the test specimen and the strain was monitored based on the movements of these marked points. A non-contact video extensometer (iMetrum Ltd, Bristol, UK) was used to monitor the strain of the test specimen tested at the lowest crosshead displacement speed of 1 mm min^−1^. At higher testing speed, a high-speed camera (Phantom, Vision Research Inc, USA) was used that captured the movements of the two marked points. A frame rate of 1,000 frames per second was used. The distance between these two marked points was post-processed using digital image correlation (DIC) into strain values. At high crosshead displacement speed, the acceleration of the crosshead will affect the measured tensile properties of the test specimen. Therefore, the test specimen was slacked (buckled) slightly to account for this acceleration such that the desired strain rate was achieved immediately when the specimen was loaded under tension. Young’s modulus was calculated from the slope of the stress strain curve in the strain interval of between 0.05% and 0.25% in accordance with BS ISO 527: 2012. Average results of 5 test specimens were reported for each strain rate and nanopaper grammage. Only specimen failed in the gauge section was considered a valid failure. All tests were performed at room temperature (22 °C) and at a relative humidity of 40%.

### Viscoelastic properties of the BC nanopapers

The viscoelastic behaviour of BC nanopapers was characterised using a dynamic mechanical analyser (RSA-G2 Solids Analyser, TA Instruments) in tension mode. Rectangular test specimen with dimensions of 35 mm × 5 mm was used. After mounting the sample onto the fixture, the test specimen had an exposed length of 20 mm. The specimens were subjected to a strain amplitude sweep from 0.05 to 0.35% at frequencies of 0.1, 1, and 10 Hz. A pre-tension value of 3 N was applied to avoid the buckling of the test specimen during the compressive part of the stress–strain cycle.

### Quantifying the creep of BC nanopapers

The creep of BC nanopapers was characterised by loading BC nanopapers at a constant load for 8 h using a micro-tensile tester (Model MT-200, Deben UK Ltd., Woolpit, UK) equipped with a 200 N load cell. The test specimen was subjected to constant uniaxial tensile stresses ranging from 30 to 90 MPa. Rectangular test specimens with an overall length of 35 mm and a width of 5 mm were used. After mounting onto the micro-tensile tester, the test specimen possessed an exposed (gauge) length of 20 mm. A crosshead displacement speed of 0.5 mm min^−1^ was employed. The strain of the test specimen was measured by a non-contact video extensometer (iMetrum Ltd, Bristol, UK). All tests were performed at room temperature (22 °C) and at a relative humidity of 40%.

## Supplementary information

Supplementary Information
