# Supplementary material for: Anomalous tensile response of bacterial cellulose nanopaper at intermediate strain rates
Source: Sci Rep. 2020 Sep 17;10:15260. doi: 10.1038/s41598-020-72153-w (PMC7498453; doi:10.1038/s41598-020-72153-w)
Supplement: Supplementary file 1 — Supplementary Information [file 41598_2020_72153_MOESM1_ESM.docx]

**Supplementary information**

**Anomalous tensile response of bacterial cellulose nanopaper at intermediate strain rates**

Alba Santmarti, Hon Wah Liu, Koon-Yang Lee*

Department of Aeronautics, Imperial College London, South Kensington Campus, SW7 2AZ, London, UK

*Corresponding author: Email (KYL): koonyang.lee@imperial.ac.uk; Tel: +44 (0)20 7594 5150; Fax: +44 (0)20 7383 2348

**List of tables and figures**


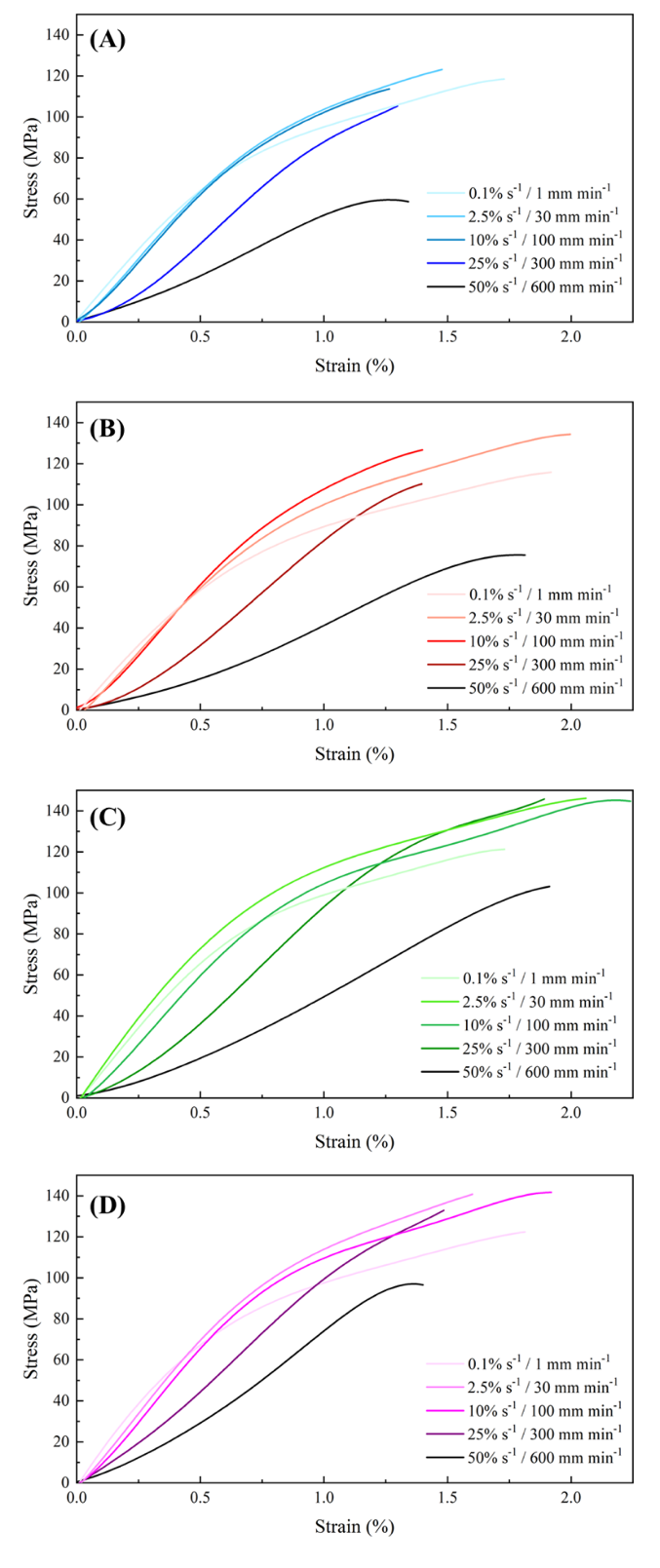


**Fig. S1. Representative stress-strain curves at different strain rents of BC nanopapers of different grammage (a) 20 gsm, (b) 40 gsm, (c) 60 gsm and (d) 80 gsm.**

**Table S1. Tensile properties of BC nanopapers with different grammages tested at different strain rates.**

| **Grammage (g m^-2^)**  **(g (g/m^2^)** | **Strain rate (% s^-1^)** | **Crosshead speed (mm min^-1^)** | **Elastic modulus (GPa)** | **Ultimate strength (MPa)** | **Strain at break (%)** | **Yield Strength (MPa)** |
| --- | --- | --- | --- | --- | --- | --- |
| 20 | 0.1 | 1 | 13.4 ± 0.9 | 113 ± 10 | 1.58 ± 0.27 | 72 ± 5 |
|  | 2.5 | 30 | 13.0 ± 1.2 | 127 ± 12 | 1.65 ± 0.26 | 84 ± 7 |
|  | 10 | 100 | 11.4 ± 2.1 | 121 ± 15 | 1.42 ± 0.39 | 92 ± 7 |
|  | 25 | 300 | 5.1 ± 0.4 | 111 ± 15 | 1.40 ± 0.24 | 96 ± 7 |
|  | 50 | 600 | 2.2 ± 1.2 | 49 ± 7 | 1.35 ± 0.11 | 45 ± 7 |
|  |  |  |  |  |  |  |
| 40 | 0.1 | 1 | 13.7 ± 1.8 | 123 ± 10 | 1.82 ± 0.35 | 76 ± 2 |
|  | 2.5 | 30 | 13.9 ± 1.4 | 137 ± 5 | 1.71 ± 0.41 | 92 ± 6 |
|  | 10 | 100 | 11.3 ± 2.0 | 132 ± 11 | 1.55 ± 0.22 | 90 ± 8 |
|  | 25 | 300 | 5.2 ± 1.5 | 118 ± 17 | 1.49 ± 0.08 | 112 ± 15 |
|  | 50 | 600 | 1.5 ± 1.0 | 52 ± 16 | 1.60 ± 0.25 | 50 ± 15 |
|  |  |  |  |  |  |  |
| 60 | 0.1 | 1 | 14.7 ± 1.0 | 121 ± 7 | 1.68 ± 0.19 | 80 ± 3 |
|  | 2.5 | 30 | 11.9 ± 2.6 | 143 ± 7 | 1.98 ± 0.09 | 98 ± 6 |
|  | 10 | 100 | 11.9 ± 1.0 | 138 ± 15 | 1.65 ± 0.36 | 103 ± 9 |
|  | 25 | 300 | 7.15 ± 0.9 | 127 ± 12 | 1.62 ± 0.18 | 106 ± 10 |
|  | 50 | 600 | 4.31 ± 0.5 | 98 ± 12 | 1.76 ± 0.16 | 95 ± 12 |
|  |  |  |  |  |  |  |
| 80 | 0.1 | 1 | 14.9 ± 0.8 | 126 ± 14 | 1.66 ± 0.14 | 75 ± 7 |
|  | 2.5 | 30 | 11.4 ± 2.2 | 137 ± 19 | 1.71 ± 0.37 | 94 ± 6 |
|  | 10 | 100 | 6.3 ± 3.7 | 143 ± 3 | 2.00 ± 0.06 | 99 ± 9 |
|  | 25 | 300 | 7.1 ± 0.9 | 133 ± 10 | 1.53 ± 0.07 | 109 ± 9 |
|  | 50 | 600 | 5.0 ± 0.8 | 92 ± 16 | 1.30 ± 0.10 | 87 ± 15 |
